# Functional Balance and Motor Impairment Correlations with Gait Parameters during Timed Up and Go Test across Three Attentional Loading Conditions in Stroke Survivors

**DOI:** 10.1155/2014/439304

**Published:** 2014-03-13

**Authors:** Haidzir Manaf, Maria Justine, Mazlifah Omar

**Affiliations:** ^1^Department of Physiotherapy, Faculty of Health Sciences, Universiti Teknologi MARA, Puncak Alam Campus, 42300 Puncak Alam, Selangor, Malaysia; ^2^Department of Rehabilitation Medicine, Faculty of Medicine, Universiti Teknologi MARA, 47000 Sungai Buloh, Selangor, Malaysia

## Abstract

The aim of this study was to determine whether stroke survivor's gait performance during dual-task Timed Up and Go (TUG) test is correlated with the level of functional balance and motor impairment. Thirty stroke survivors (22 men, 8 women) were recruited for this study. The level of functional balance (Berg Balance Scale) and motor impairment (Fugl-Meyer assessment lower extremity) were assessed prior to the TUG test. TUG test was conducted under three attentional loading conditions (single, dual motor, and dual-cognitive). The time and number of steps were used to quantify gait parameters. The Spearmen's rank correlation coefficient was used to evaluate the relationship between these variables. There was moderate to strong negative correlation between functional balance and gait parameters (range −0.53 to −0.73, *P* < 0.05). There was a weak negative correlation observed between the time taken to complete the single task and motor impairment (*r*_*s*_ = −0.43; *P* = 0.02) dual motor task and motor impairment (*r*_*s*_ = −0.41; *P* = 0.02). However, there were no significant correlations between lower limb motor impairment and the number of steps in all conditions. These findings suggest that functional balance may be an influential domain of successful dual-task TUG in stroke.

## 1. Introduction

The incidence of falls is one of the most common consequences of stroke, especially when it involves walking in the community or outdoors [1, 2]. Walking outdoors is a challenging task for stroke survivors because they need to overcome obstacles and change direction (turning) under the presence of many distractions [3] and at the same time perform multiple tasks simultaneously [4]. In order to be able to adapt to this challenging environment, stroke survivors may be required to learn highly complex skills [4] such as walking and turning as well as performing dual task such as walking while carrying objects or answering phone calls.

Skilled turning has been identified as one of the influential factors for safe functional ambulation [5–7]. Turning sequence involves slowing down the walking speed as well as reorientation of the body segments toward the new direction [8, 9]. It is postulated that stroke survivors with impaired sensorimotor, balance, and cognitive or attentional function may have difficulty performing safe turning task.

The Timed Up and Go test (TUG), which is a clinical assessment for the balance and mobility performance [10], has been highly used in a clinical setting to represent functional balance by measuring the time taken for an individual to stand up from a chair, walk 3 meters, turn around, walk 3 meters back to the chair, and sit down [11]. It is also a reliable tool to evaluate agility because it involves walking and a change of direction [7]. In other words, the TUG test can be used to represent the ability to walk and turn 180 degrees. The time taken to complete the TUG test has been shown to predict falls in community-dwelling elderly and stroke survivors [12, 13]. If an individual takes more than 5 steps or more than 3 seconds to complete the turning during the TUG test, he/she is considered as having turning difficulty [14]. In another study, the TUG scores were shown to be able to differentiate between stroke survivors and healthy elderly and correlated well with plantar flexor strength, gait performance, and walking endurance in chronic stroke survivors [13]. Similarly, a study has shown that stroke survivors with falls history took significantly longer time to turn than age-matched controls, but no kinematic differences were found that could contribute to falls history or falls risk [7]. These reports indicate that a further investigation is required in order to assess other associated factors that may correlate well with turning performance especially turning with additional tasks.

A previous study had found that performing TUG test under dual-task condition increased the time taken to complete the task and the effect was greater in elderly with a history of falls than those without [12]. In the community-dwelling elderly, the deterioration of gait performance during single and dual-task TUG was influenced by their balance performance [15, 16]. However, to date, little attention has been paid to examining the relationships between balance performance and dual-task TUG in stroke survivors. Exploration of these associations is important for guiding the development of intervention for stroke survivors who have a high risk of falls while at the same time trying to encourage functional ambulation especially in the community setting.

Therefore, this study has 2 specific aims: firstly, to evaluate the relationship between balance performance and gait parameters and secondly, to examine the relationship between lower limbs motor impairment and gait parameters across three attentional loading conditions (single, dual motor, and dual-cognitive task conditions) during turning as indicated by the Timed Up and Go (TUG) test in stroke survivors. The sensitivity of the TUG test might be able to capture the effects of dual-task conditions on gait performance during turning while walking. Dual task refers to the ability to carry out more than one task at the same time [17]. We predicted that balance performance would correlate significantly with gait parameters across the three attentional loading conditions. We also hypothesized that the level of lower limb motor impairment demonstrates a weak correlation with the same parameters.

## 2. Methods

### 2.1. Participants

A total of 30 stroke survivors (22 males, 8 females) participated in this study. Among 30 participants, 17 presented with left sided hemiparesis. Participants were recruited from the government funded hospital using a purposive sampling. Stroke survivors were included if they met the following criteria: (1) at least 6 months after stroke as diagnosed by a medical physician, (2) ability to walk continuously for 10 meters independently without walking aid, (3) ability to hold a glass full of water with the nonaffected hand, (4) able to follow 3 steps command, and (5) ability to do simple arithmetic. Participants were excluded if they had more than one stroke, other neurologic disorders (e.g., Parkinson's disease and traumatic brain injury), and severe orthopedic conditions (e.g., joint deformities, osteoarthritis, and rheumatoid). They were also excluded if they had visual field defects or scored less than 24 in the Mini-Mental State Examination (MMSE). The study was approved by the institutional ethics committee, and participants signed written informed consent prior to participation.

### 2.2. Outcome Measures

This is a cross-sectional study design. We measured participants' cognitive function with MMSE [18], stroke severity with Fugl-Meyer lower extremity assessment (FMLE) [19], and functional balance with Berg Balance Scale (BBS) [20]. The FMLE is a valid and reliable measure of motor recovery following a stroke. It consists of 17 items for lower extremity subscale, with each item scored on a 0–2 scale (score range, 0–34) [21]. Meanwhile, BBS is used as a clinical test of a person's ability to maintain balance. BBS is a 5-point scale (0–4) with a maximum total score of 56 (higher scores indicate better balance) that evaluates functional balance in 14 different activities [20]. The BBS demonstrates good correlation with laboratories and clinical measures in relation to instability and falls in various populations [22]. It has been demonstrated to have a strong correlation with TUG among the elderly [23] and stroke survivors [5]. In addition, the time taken to complete the dual-task (motor and cognitive) TUG test has a strong correlation with BBS in the community-dwelling elderly [16].

### 2.3. Procedures

**Figure 1:**
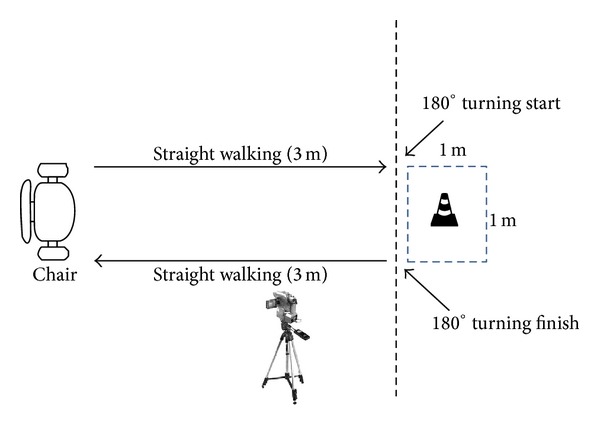
Diagrammatic presentation of Timed Up and Go test.

The study was conducted in a physiotherapy gymnasium that was equipped with bright lighting and even flooring. The turning area for the TUG test (1 meter by 1 meter) was marked on the floor to indicate the direction for participants to turn around (Figure 1). Data were collected using a calibrated digital stopwatch and digital video camera (30 frames per second). A 5-minute rest was given between tests to minimize any fatigue effects. Participants performed three practice trials to familiarize themselves with the test before implementing the real trial. The test consisted of 3 conditions in the following order.

*(i) Single Task TUG.* During a single task condition, stroke survivors performed the TUG test only (without a secondary task). A standard armchair was used, and a cone was placed at the 3-meter mark of the walking path. Participants sat comfortably with their back against the chair and the researcher gave the instruction as follows. “When I say “Go,” please stand up from the chair and walk to the cone; turn to your right/left after you pass the cone, walk back, and sit down on the chair; please walk at your comfortable speed.” We asked the participants to turn towards the paretic side for 3 trials.

*(ii) Dual Motor Task TUG.* For the dual motor task condition, participants sat comfortably with their back against the chair while holding a glass full of water with their nonaffected hand [12]. When the researcher said “Go,” they stood up from the chair, walked 3 meters with a comfortable speed, turned 180° towards the paretic side, walked 3 meters back to the chair, and sat down. This was repeated for 3 trials. During the testing, participants were instructed to hold the glass without spilling the water (prioritized glass holding) or else the trial would be considered as a failed trial and need to be repeated. On average, 16.7% of trials were repeated.

*(iii)  Dual Cognitive Task TUG.* Participants sat comfortably with their back against the chair. The researcher verbally gave them a number (any number from 20 to 100). The participants then counted backwards by three from the number consecutively and gave verbal responses. For example, if the given number was 100, participants responded as follows: “97, 94, 91,….” When they heard “Go”, they stood up from the chair, walked 3 meters with a comfortable speed, turned 180° towards the paretic side, walked 3 meters back to the chair, and sat down. This was repeated for 3 times. Trials that were considered failed needed to be repeated. This may happen when participants made error on the counting backward task. On average, 33.3% of trials were repeated.

### 2.4. Statistical Analysis

Nonparametric statistics were used to conduct statistical analysis using the IBM SPSS statistical software version 19 (IBM SPSS, Armonk, NY). We measured the gait performance (number of steps and time taken) to complete the TUG test. Our previous work [24] had found that turning direction (towards paretic or nonparetic side) did not have an effect on the TUG performance in both stroke survivors and their matched peers. Therefore, we analyzed participants' gait performance during turning towards the paretic side.

The median and range were presented as descriptive statistics. The Friedman test was used to determine whether stroke survivors would take a greater number of steps and longer time to complete the TUG test under the attentional loading conditions (dual motor and dual-cognitive) compared to a single task condition. Spearman's rank-order correlation coefficients (*r*_*s*_) were used to identify the associations between the levels of functional balance, lower limbs motor impairment, and gait parameters (number of steps and time taken) across three attentional loading conditions. The strength of the relationship between the variables was based on Portney and Watkins's guidelines; a correlation from 0.00 to 0.25 indicates small or no relationship, between 0.25 and 0.50, a small degree of relationship, and between 0.50 and 0.75, a moderate to a good degree of relationship; values above 0.75 are taken into consideration for having an excellent degree of relationship [25]. The significance level for all analyses was set at 0.05.

## 3. Results

Participant's characteristics were presented in Table 1. Among the 30 stroke survivors, 17 presented with left hemiparesis.

### 3.1. Effects of Attentional Loading on Gait Parameters

**Figure 2:**
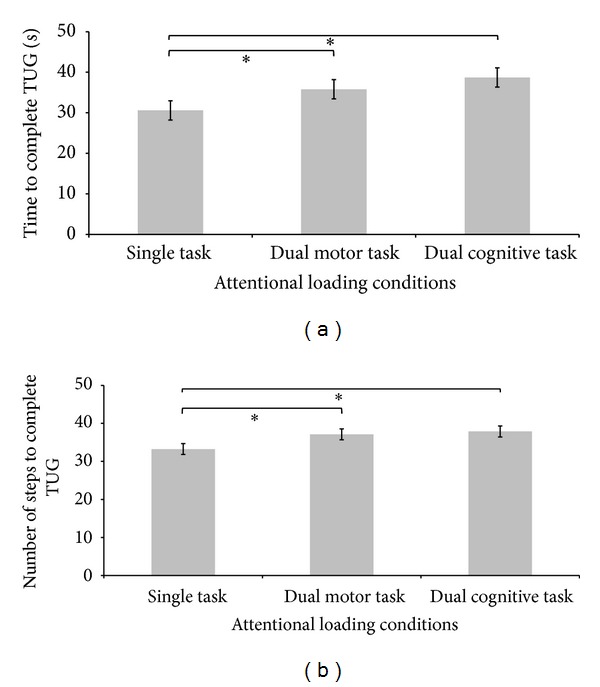
Comparison of gait performance under three attentional loading conditions in stroke survivors. (a) Time to complete TUG. (b) Number of steps to complete TUG. Asterisks (*) signify statistically significant differences (*P* < 0.05). Error bars represent standard error.

Figure 2(a) shows the time taken to complete the TUG test under the three attentional loading conditions in stroke survivors. Friedman's test shows a statistically significant increase in time taken to complete TUG (*X* ^2^ = 20.8, df = 2, and *P* = 0.001). Post hoc analysis with Wilcoxon signed-rank test shows that TUG time in dual motor is significantly longer than the single task condition (*Z* = −3.43, *P* = 0.001). A significant longer TUG time was seen in dual-cognitive than single task condition (*Z* = −4.06, *P* = 0.001), while the difference between dual motor and dual-cognitive conditions did not reach significance level (*Z* = −1.84, *P* = 0.07).

Figure 2(b) shows the number of steps to complete the TUG test under the three attentional loading conditions in stroke survivors. The application of Friedman's test shows a statistically significant increase in the number of steps to complete TUG test (*X* ^2^ = 16.2, df = 2, and *P* = 0.001). Post hoc analysis with Wilcoxon signed-rank test shows that the number of steps to complete TUG test in dual motor is significantly higher than single task condition (*Z* = −3.86, *P* = 0.001). A significant greater number of steps taken to complete TUG test were also seen in dual-cognitive than single task condition (*Z* = −3.5, *P* = 0.001), while the difference between dual motor and dual-cognitive conditions did not reach significance level (*Z* = −6.64, *P* = 0.53).

### 3.2. Correlation between BBS and Gait Parameters

There was a strong negative rank correlation between the time taken to complete the single task condition and BBS (*r*_*s*_ = −0.72; *P* < 0.01). There was a moderate negative rank correlation between the time taken to complete the dual motor task conditions and BBS (*r*_*s*_ = −0.67; *P* = 0.001). In addition, there was a moderate negative rank correlation between the time taken to complete the dual-cognitive task conditions and BBS (*r*_*s*_ = −0.53; *P* = 0.003).

**Figure 3:**
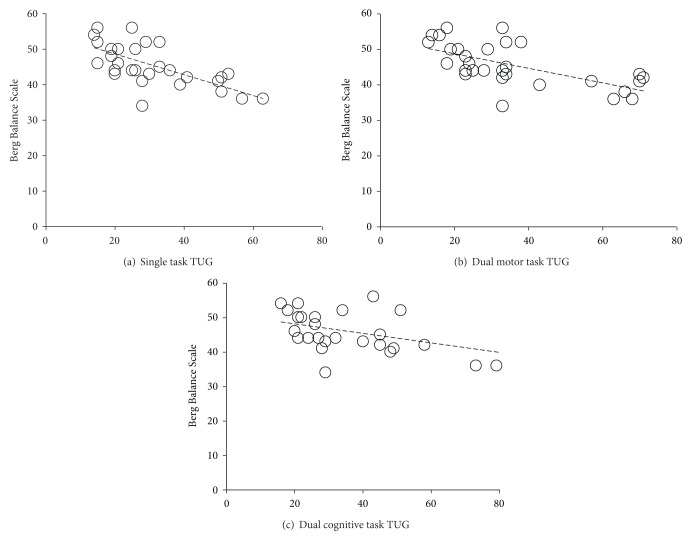
Scatter plots between Berg Balance Scale (BBS) scores and time to complete Timed Up and Go (TUG) test across three attentional loading conditions. (a) Single task TUG, (b) dual motor task TUG, and (c) dual cognitive task TUG.

**Figure 4:**
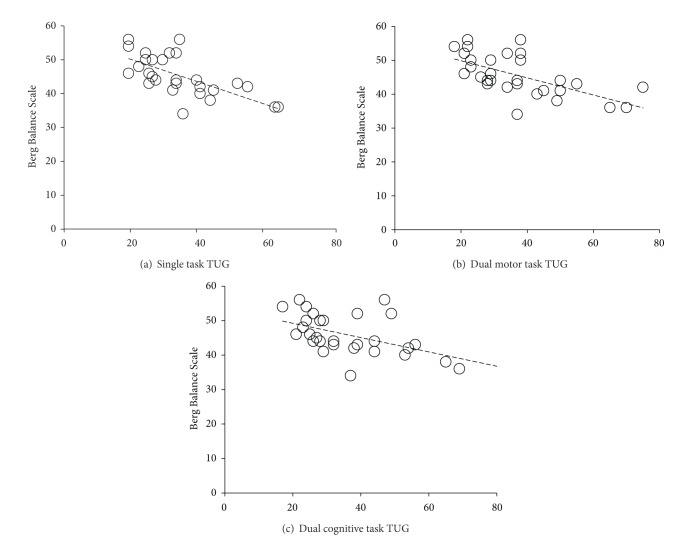
Scatter plots between Berg Balance Scale (BBS) scores and number of steps to complete Timed Up and Go (TUG) test across three attentional loading conditions. (a) Single task TUG, (b) dual motor task TUG, and (c) dual cognitive task TUG.

In terms of the number of steps, there was a strong negative rank correlation between the numbers of steps to complete the single task condition (*r*_*s*_ = −0.73; *P* = 0.001). There was a moderate negative rank correlation between number of steps to complete dual motor task condition (*r*_*s*_ = −0.65; *P* = 0.001), dual-cognitive task condition (*r*_*s*_ = −0.61; *P* = 0.001), and BBS. The scatter plots for each task condition are presented in Figures 3 and 4.

### 3.3. Correlation between FMLE and Gait Parameters

**Figure 5:**
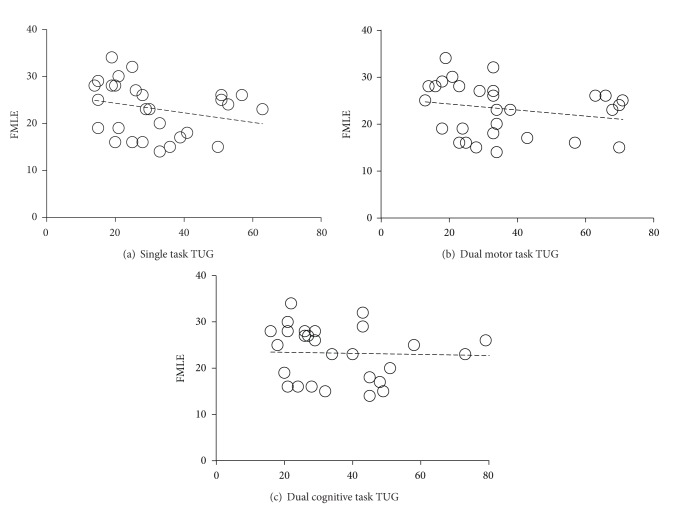
Scatter plots between Fugl-Meyer lower extremity motor (FMLE) scores and time to complete Timed Up and Go (TUG) test across three attentional loading conditions. (a) Single task TUG, (b) dual motor task TUG, and (c) dual cognitive task TUG.

**Figure 6:**
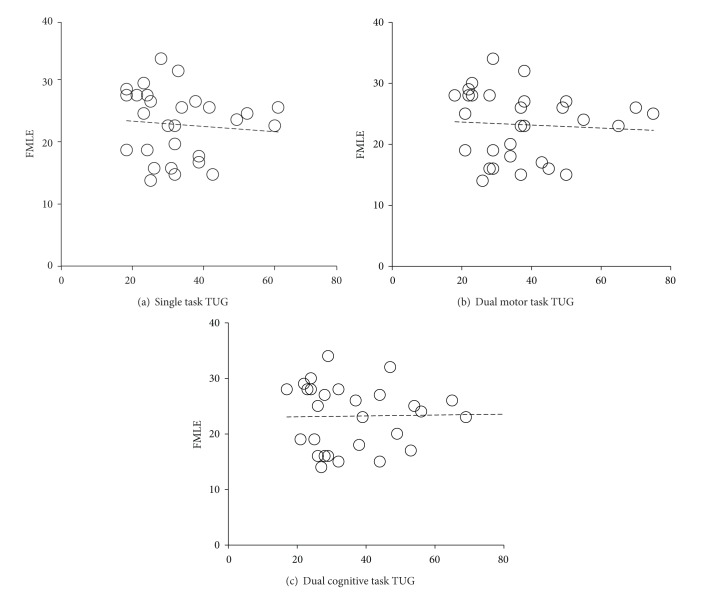
Scatter plots between Fugl-Meyer lower extremity motor (FMLE) scores and number of steps to complete Timed Up and Go (TUG) test across three attentional loading conditions. (a) Single task TUG, (b) dual motor task TUG, and (c) dual cognitive task TUG.

There was a weak negative correlation between FMLE and the time taken to complete the single task (*r*_*s*_ = −0.43; *P* = 0.02) and dual motor task condition (*r*_*s*_ = −0.41; *P* = 0.03). However, no significant correlation was found in the dual-cognitive task conditions (*P* > 0.05). For the number of steps, there was no significant correlation with FMLE across single, dual motor, and dual-cognitive task conditions (*P* > 0.05). The scatter plots for each task condition are presented in Figures 5 and 6.

## 4. Discussion

The correlation between balance performance, motor impairment, and gait parameters during TUG test in single task manner has been reported previously [26, 27]. Little is known whether functional balance and motor impairment have influences on gait performance under different attentional loading conditions during TUG. In this study, the relationship between the functional balance, lower limb motor impairment and gait parameters (number of steps and time taken) during TUG across different attentional loading conditions (single, dual motor, and dual-cognitive) in stroke survivors was examined. We presented a few important findings. First, attentional loading led to deterioration of gait performance. Second, we found that functional balance was significantly correlated with gait parameters during single, dual-motor, and dual-cognitive task turning. Lastly, although lower limb motor impairment was significantly correlated with the time taken during single and dual motor task, no significant correlation was seen in the other variables.

Theoretically, the deterioration of gait performance can be explained by several neurophysiological theories such as capacity sharing, bottleneck, and multiple theories [28]. The results of our study may be best explained by the capacity sharing model of dual-task interference. The capacity sharing theory assumes that the attentional resources are fixed in capacity while the task performance depends on the amount of resources that is allocated to the task [28]. A performance task like walking requires some amount of attentional resources. Dual-task interference is expected to be seen if the available attentional resource capacity is exceeded, thus provoking a performance reduction in either one task or both tasks depending on task prioritization approach [29]. In this study, participants were asked to prioritize the secondary task (dual motor and dual-cognitive task). Participants were instructed to hold the glass without spilling the water (dual-motor) and performed counting as accurately as they could. As a result, participants demonstrated deterioration in gait performance including longer time and greater number of steps to complete TUG test.

Interestingly, we found that functional balance has moderate to strong negative correlation with gait parameters during single, dual-motor, and dual-cognitive task conditions. In other words, stroke survivors who showed lower scores on BBS presented with a longer time and a higher number of steps when they were required to perform two tasks simultaneously. This finding corroborates the ideas of Hofheinz and Schusterschitz [16] who found the time taken to complete the TUG test across different attentional conditions (dual motor and dual-cognitive) has strong correlations with BBS (*r*, range between −0.66 and −0.72) in the elderly. This result may be explained by the fact that both outcomes are assessing the same functional balance domain [6]. The TUG test is used to assess functional balance in various populations. The time taken to complete the TUG test demonstrates a strong correlation with laboratory measurement [30] and clinically based assessment such as BBS. However, these tests are not challenging enough to identify fallers when applied to ambulatory stroke survivors. For this reason, evaluation of attention using dual-task paradigm could be useful to indicate factors that may contribute to fall. This evidence could explain the relationship between TUG and BBS especially when the task becomes more challenging, such as an addition of a secondary task (motor and cognitive). Another possible explanation for this is that TUG test requires stepping and turning activity which involves shifting the centre of gravity that in turn challenges balance performance, especially in stroke survivors. Functional balance performance was reported as a significant predictor of falling [31] and patients who experienced at least one fall showed significantly lower scores on BBS [32]. Therefore, it can be concluded that functional balance is an essential domain that may influence successful turning while walking with dual-task conditions for individuals with stroke.

We also found that functional balance was significantly highly correlated with gait performance during the single task condition only. The results of the current study might also have been implicated by the variability in the gait performance. Additional tasks may increase the variability in gait performance, and it would be influenced by the degree of difficulty. This finding further supports the idea that the attentional resources are fixed in capacity and deterioration of performance will be observed in either one task or both tasks [28]. In this study, the deterioration was observed in gait performance as participants were asked to prioritize secondary task (motor and cognitive).

As expected, a weak negative correlation was found between lower limbs motor impairment and the time taken to complete the single and dual motor task condition. It is comparable to a previous study that low to moderate correlation was found between lower limb strength and the TUG test performance (*r* = −0.33 to −0.64) [27]. In addition, the FMLE has been also shown to present with a weak association with falls history while the BBS demonstrated fair accuracy in identifying people with multiple falls with a cut-off score of 49 and a positive logistic regression of 2.80 [33]. However, no significant correlation was found between the lower limb motor impairment and the time taken to complete the dual-cognitive task condition. The difficulty of cognitive task was found to cause some of the participants to compromise the cognitive task performance for stability. Furthermore, there was no significant correlation between lower limb motor impairment and number of steps in the three attentional loading conditions. This finding indicates that a lower score in FMLE was not accompanied by an increased number of steps to complete TUG. It seems possible that these results are due to the adoption of a stepping strategy in which participants slowed down the gait speed and took a longer pausing duration between steps. The pauses between steps may explain why the time taken to complete turning increases during the dual motor task while the number of steps remains the same. Therefore, it is possible to conclude that the motor impairment of the lower limbs does not have a central role in gait performance during TUG test under dual-task conditions.

In summary, this study has demonstrated the existence of relationships between functional balance and gait parameters across three attentional loading conditions during turning while walking in stroke survivors. We noted several limitations of this study. First, we did not control the trade-off between the walking task and the counting backward task. Some of the participants compromised the cognitive task performance for stability. Second, some of the participants were asked to repeat failed trials that may cause training effects. Third, this study only included stroke survivors but not age-matched controls. Thus, it is inconclusive whether the gait deterioration under the dual-task condition was a result of stroke or aging. It is recommended that future studies compare stroke survivors to healthy age- and gender- matched controls. Lastly, future study should include information about stroke lesion and size of the lesion in order to identify crucial brain areas involved in gait performance during the TUG test.

Stroke survivors with impaired functional balance presented with greatest deterioration of gait parameters across all task conditions such as a longer time and a greater number of steps to perform the dual task turning. The ability to carry out dual tasks after stroke is crucial to ensure a safe and effective gait especially in outdoor settings. Thus, it is recommended that dual-task TUG test be considered as an alternative assessment to enhance gait recovery among patients with stroke.

## Figures and Tables

**Table 1:** General characteristics of stroke survivors and healthy control participants.

Participant characteristics	Median	Range
Age (year)	27.0	36–80
Poststroke duration (month)	12.0	6–32
Body weight (kg)	66.8	45–93
Body height (meter)	1.68	1.58–1.85
Mini-Mental State Examination (MMSE)	30.0	24–30
Berg Balance Scale (BBS)	44.0	34–56
Fugl-Meyer lower extremity	24.5	14–34
